# A Novel Clinician-Orchestrated Virtual Reality Platform for Distraction During Pediatric Intravenous Procedures in Children With Hemophilia: Randomized Controlled Trial

**DOI:** 10.2196/10902

**Published:** 2019-01-09

**Authors:** Amy Dunn, Jeremy Patterson, Charmaine F Biega, Alice Grishchenko, John Luna, Joseph R Stanek, Robert Strouse

**Affiliations:** 1 Nationwide Children's Hospital Division of Hematology, Oncology and BMT The Ohio State University Columbus, OH United States; 2 The Research Institute at Nationwide Children’s Hospital Columbus, OH United States; 3 The Ohio State University Advanced Computing Center for the Arts and Design Columbus, OH United States; 4 Nationwide Children’s Hospital Division of Hematology/Oncology/BMT Columbus, OH United States

**Keywords:** anxiety, distraction, hemophilia, intravenous, mobile phone, needle, pediatric, virtual reality

## Abstract

**Background:**

Needles are frequently required for routine medical procedures. Children with severe hemophilia require intensive intravenous (IV) therapy to treat and prevent life-threatening bleeding and undergo hundreds of IV procedures. Fear of needle-related procedures may lead to avoidance of future health care and poor clinical outcomes. Virtual reality (VR) is a promising distraction technique during procedures, but barriers to commercially available VR platforms for pediatric health care purposes have prevented widespread use.

**Objective:**

We hypothesized that we could create a VR platform that would be used for pediatric hemophilia care, allow clinician orchestration, and be safe and feasible to use for distraction during IV procedures performed as part of complex health care.

**Methods:**

We created a VR platform comprising wireless, adjustable, disposable headsets and a suite of remotely orchestrated VR games. The platform was customized for a pediatric hemophilia population that required hands-free navigation to allow access to a child’s hands or arms for procedures. A hemophilia nurse observing the procedure performed orchestration. The primary endpoint of the trial was safety. Preliminary feasibility and usability of the platform were assessed in a single-center, randomized clinical trial from June to December 2016. Participants were children with hemophilia aged 6-18 years. After obtaining informed consent, 25 patients were enrolled and randomized. Each subject, 1 caregiver, and 1 hemophilia nurse orchestrator assessed the degree of preprocedural nervousness or anxiety with an anchored, combined modified visual analog (VAS)/FACES scale. Each participant then underwent a timed IV procedure with either VR or standard of care (SOC) distraction. Each rater assessed the distraction methods using the VAS/FACES scale at the completion of the IV procedure, with questions targeting usability, engagement, impact on procedural anxiety, impact on procedural pain, and likability of the distraction technique. Participants, caregivers, and nurses also rated how much they would like to use VR for future procedures. To compare the length of procedure time between the groups, Mann-Whitney test was used.

**Results:**

Of the 25 enrolled children, 24 were included in the primary analysis. No safety concerns or VR sickness occurred. The median procedure time was 10 (range 1-31) minutes in the VR group and was comparable to 9 (range 3-20) minutes in the SOC group (*P*=.76). Patients in both the groups reported a positive influence of distraction on procedural anxiety and pain. Overall, in 80% (34/45) of the VR evaluations, children, caregivers, and nurses reported that they would like to use VR for future procedures.

**Conclusions:**

We demonstrated that an orchestrated, VR environment could be developed and safely used during pediatric hemophilia care for distraction during IV interventions. This platform has the potential to improve patient experience during medical procedures.

**Trial Registration:**

Clinical Trials.gov NCT03507582; https://clinicaltrials.gov/ct2/show/NCT03507582 (Archived by WebCite at http://www.webcitation.org/73G75upA3)

## Introduction

Medical procedures involving needles induce fear in most people, especially children [1-6]. However, treatment for some medical conditions requires frequent needle sticks. Balancing the treatment needs for life-threatening conditions with the fear and anxiety imposed by the mechanism of these treatments is an important challenge for the medical community. In addition to the load needle sticks impose on patients and families, the medical system and medical providers also bear burden and expenses related to needles. One of the conditions that requires the most needle-intensive care is hemophilia. Both hemophilia A (HA) and B (HB) are severe congenital bleeding disorders. Without intravenous (IV) infusions of clotting factor concentrates, children with hemophilia experience life- and limb-threatening bleeding. Most children with hemophilia begin routine IV infusions of factor concentrate between the age of 1 and 3 years and continue infusions 2-3 times per week for life. This translates to thousands of necessary needle sticks over the course of their lives and a high treatment burden related to needles [7]. Needle fear related to IV procedures, particularly in children with hemophilia, can progress to blood-injection-injury phobia (needle phobia), treatment avoidance, and poor adherence, all of which can contribute to poor medical outcomes [8]. Psychological interventions, such as distraction and hypnosis, can reduce needle-related pain and distress in pediatrics [9]. A recent systematic review and meta-analysis demonstrated that distraction could lead to reductions in child- and observer-reported pain and distress during needle procedures. However, among the major elements of distraction, the relative importance of (1) no or low-tech versus high-tech distracters; (2) child engaging versus passive distracters; (3) degree of adult involvement; and (4) the availability of child choice in the success of these distracters is unclear [10]. Specific virtual reality (VR)-based distractions have shown promise in clinical settings, such as dentistry, IV placement, occupational or physical therapy, and burn care [9,11-24]. In particular, VR approaches have demonstrated decreased pain associated with dental procedures, physical therapy, and burn dressing changes. Although various consumer-grade VR technologies are available, most carry barriers that prevent clinical implementation [11-18,20-24]. Cost is another barrier, with the top-selling commercial unit having an approximate cost of US $500. This includes the head-mounted display (HMD), wireless controllers, and proximity sensors, but not a personal computer capable of running VR content, with a cost of approximately US $2000. On the low end, VR content is available for smartphones and low-cost HMDs, at around US $800 for the smart device and approximately US $35 for HMD. In either case, these estimates do not include the cost of content or technical support to configure and maintain the systems. Additionally, the unit is tethered to the personal computer and, as a result, cannot easily move between locations. Infection concerns exist with nondisposable headsets particularly in a pediatric setting, such as a hematology or oncology clinic. Additionally, most commercial VR platforms do not allow a medical provider to direct or modify the VR environment in response to patient distress and also focus on the experience of patients and not of caregivers.

Despite the barriers to VR implementation described above, we hypothesized that a children’s hospital-based team with expertise in hemophilia patient care and user experience technology could create a safe and clinically feasible VR ecosystem that met the needs of patients, caregivers, and medical providers during IV procedures. Assessing safety and testing the feasibility of the designed ecosystem in a complex clinical environment were the goals of the project. A comprehensive hemophilia clinic visit was selected to test the safety, feasibility, and likeability of the ecosystem. During these visits, patients see multiple providers in addition to having IV procedures, so the efficiency of clinic flow is vitally important. We specifically designed the ecosystem for children with hemophilia because patients with hemophilia need a frequent distraction from needles safe and successful integration into a complex clinic environment would demonstrate the likelihood of feasibility in less complex situations.

## Methods

### Virtual Reality Platform Design

**Figure 1:**
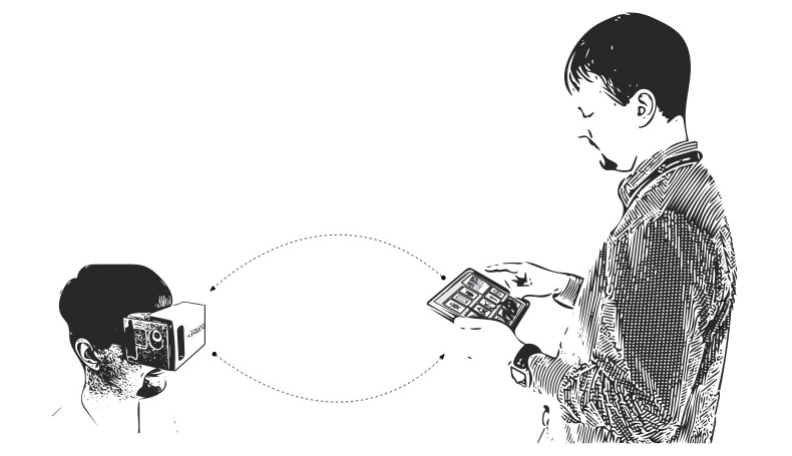
Manual orchestration of child engaging events in a virtual reality environment from a connected virtual digital interface from an embedded viewpoint.

The VR platform design team consisted of the Nationwide Children’s Hospital Hemophilia Treatment Center (HTC) staff and the user experience technology team. The HTC staff included a hematologist, nurse clinicians, a social worker, and a psychologist. The user experience team included a project lead, industrial designer, and 2 game designers, 1 of which also served as the illustrator and visual designer. The team designed the platform for boys and girls aged 6-18 years with HA or HB. Ideal features were identified through reoccurring meetings, which included observation of HTC visits and IV procedures. Specific platform design needs were identified. First, the cost of the overall system needed to be low. The system needed to be technically easy to implement and maintain for the nursing team. Games needed to be pediatric friendly but of sufficient quality to maintain the interest of patients and families. Additionally, the video content needed to limit the possibility of VR sickness. Importantly, the majority of IV infusions require that the medical team has easy access to the hands and arms of a patient. This meant that game control mechanics needed to be hands free and help ensure the patient remained still. Because medical care is fluid and each patient experience is unique, the design team needed to allow the medical team to orchestrate the patient experience in the real-time to meet individual needs. We also sought ways to include caregivers in the game experience because parental anxiety around IV procedures can contribute to poor outcomes, and family-centered care is an important tenet of pediatric care. The team was also mindful of the need of the system to minimize the risk of transmitting infections agents from any game component. Each element of the ecosystem—the HMD, games, and the orchestration—was refined with input from volunteer pediatric hemophilia nurse clinicians, pediatric hematologists, and children without hemophilia. The ecosystem components were also demonstrated at hemophilia community events, and patients with hemophilia and their families gave feedback to the design team. The final VR intervention used for the clinical trial consisted of custom, cordless, multisized, disposable headsets, which enabled the use of VR through iPod Touch, and immersive custom games with hands-free navigation. Navigation techniques included head glances and breath. The mechanism for nurse orchestration (Figure 1; Multimedia Appendix 1) was software running on an iPad dashboard that wirelessly communicated to iPod Touch. The orchestration dashboard offered a suite of tools allowing a nurse to respond to patient needs by deploying mini-games or providing relocation to a new setting in the VR ecosystem. The dashboard also allowed parents to monitor their child’s progress. Moreover, an expert practitioner could observe patient state and, upon noticing stress, could trigger an intervention to act as a distraction method. The system’s communication platform utilized an internal server as a relay between the dashboard and the iOS device, which ran VR activities. All signal messages passed through the intermediary system and were logged and tracked against the identifier for the orchestrated session and the type of orchestration command. Message types recorded included commands to enter or exit a mini-game, transport the patient to a virtual location, connect to a paired iOS device, adjust the volume, open the video feed from the iOS device’s camera, and calibration of the headset units for fit and viewing preference. After finalization of the platform, safety and feasibility of platform integration into a routine comprehensive HTC visit were explored. Comprehensive visits included care from multiple providers during a specified timeframe, and anything that impeded clinic flow was judged not feasible. The complexity of the care for patients with hemophilia is reflected in the number and diversity of providers available in each HTC clinic. Patients are seen for comprehensive care visits once or twice per year and during those visits are seen by numerous providers based on individual needs. This can include hematologists, nurse clinicians, research nurses, psychologists, social workers, physical therapists, nutritionists, orthopedists, dentists, genetic counselors, radiologists, advocacy coordinators, and phlebotomy staff. For the pilot study, the length of the IV procedure time was chosen as a surrogate marker of feasibility.

### Study Design, Patients, and Randomization

A prospective, monocentric, unblinded, randomized clinical trial was performed (Clinical Trials.gov NCT03507582). Multimedia Appendix 1 provides details of the trial protocol. After Institutional Review Board approval, children with HA or HB, aged 6-18 years, who were cared for in our HTC were screened for eligibility. Eligible patients had to be seen for a comprehensive HTC visit defined as seeing >3 specialty hemophilia providers during one visit and have a clinically indicated IV laboratory draw or factor infusion. Patients seen between July 1 and December 31, 2016, were eligible for participation if they and their parents or guardians could speak and write in English. Patients who were unable to use the VR equipment (visual, cognitive, or hearing impairment that precluded engagement with the VR environment), who had a known history of severe motion sickness, or uncontrolled seizures were not eligible (n=1). Those who met screening criteria were approached for participation (n=25). A hemophilia nurse obtained paper informed consent and age-appropriate assent. Enrolled patients were block randomized by computer-generated random allocation to study groups using a prespecified seed. The nurse performing randomization was blinded to group allocation. Recruited participants were randomly assigned using a 2:1 ratio to VR or standard of care (SOC) distraction with a stratified block design to maintain age strata of 12 patients aged 6-12 years and 12 patients aged 13-18 years. SOC distraction was any technique routinely available in the hematology clinic that did not include VR (ie, smart devices, bubbles, and videos).

### Study Intervention

**Figure 2:**
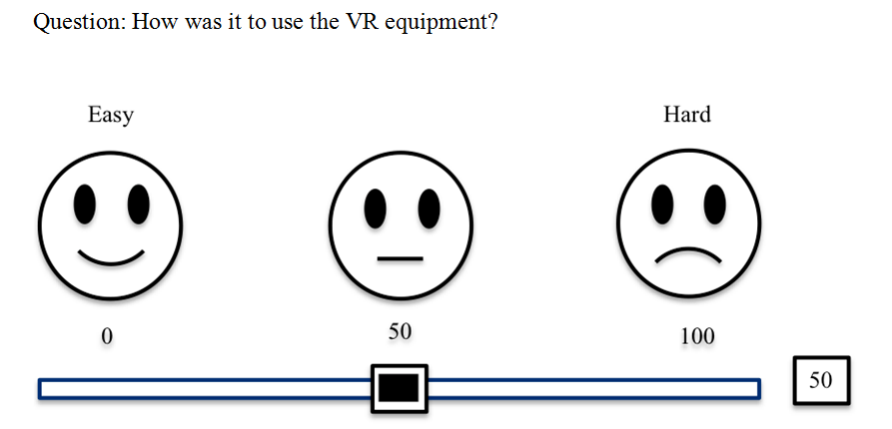
An example of the modified visual analog/FACES scale. VR: virtual reality.

All enrolled patients had a clinically indicated IV procedure. A procedure timer and survey instruments were incorporated into the orchestration iPad. A modified visual analog (VAS)/FACES (Figure 2) rating scale was used to assess the distraction techniques. The scale did not undergo specific psychometric testing; however, the FACES scale is routinely used in our hemophilia clinic. A unipolar, horizontal scale was employed to increase understandability, uniformity, and sensitivity [25-27]. All participants, caregivers, and nurses (raters) were educated in how to use the VAS/FACES scale prior to beginning the study procedure by sliding a bar below the scale picture in response to an anchored question. Raters were not blinded to participant’s distraction group, and each rater answered the study questions independently. Immediately before the IV procedure, children in the VR group were introduced to the headset, game options, and navigation techniques. Then, before being positioned for the procedure, each patient, a caregiver with them for the procedure, and a hemophilia nurse assessed the participant’s level of preprocedural anxiety using a (VAS)/FACES scale answering the question: How worried or nervous is or are you or your child or your patient about the IV procedure? Sliding the bar to the left (toward 0) represented a low degree of preprocedural worry or nervousness and to the right (toward 100) represented the highest degree of nervousness or worry. The terms nervous and worry were substituted for anxiety owing to the young age of many participants. After being positioned for the procedure, patients in the SOC group used whatever distraction they preferred, whereas patients in the VR group put on the headset, launched the game of their choice, and explored the VR environments. The nurse clinician was located next to the patient and deployed VR orchestration tools as she deemed necessary. The guardian was present throughout in both the groups. The study methodology was not altered during the trial. No revisions to the VR platform were performed or required during the trial.

### Primary Outcome

The primary aim of the study was to assess safety concerns and feasibility of integration of the VR platform into a comprehensive HTC visit. Safety concerns included discomfort from HMD, infection, and VR sickness. Barriers to feasibility that were assessed included technical issues with the set-up or orchestration. The primary surrogate marker of feasibility was the duration of the IV procedure in both study groups. Procedure time was the length of time from the moment a patient was positioned for the procedure to the completion of the procedure, and the primary and secondary outcomes did not change during the course of the trial. No VR platform performance issues or unexpected events related to the platform were encountered during the trial.

### Secondary Outcomes

#### Effectiveness of the Distraction Technique

Both patient groups assessed the effectiveness of their distraction techniques following the procedure by answering 3 questions as follows. (1) “Did the distraction technique keep you/your child /your patient engaged?” The anchors were 0=It really kept them engaged and 100=It really did not keep them engaged; (2) “Do you think the distraction technique/s changed you/your child/your patient’s nervousness/anxiety level during the IV procedure?” The anchors were 0=it decreased nervousness/anxiety a lot and 100=it increased nervousness/anxiety a lot; (3) “How did the distraction technique/s affect pain during your/your child/your patient’s IV procedure?” The anchors were 0=it made pain a lot better and 100=it made pain a lot worse.

#### Usability and Likeability

For patients randomized to the VR arm, data on the use of the VR equipment were recorded by the nurse orchestrator at the end of each procedure. Data were categorized if a participant wore the VR equipment: (1) during the entire procedure; (2) part of the procedure; or (3) only prior to the procedure. In addition, participants were asked to rate the usability of the VR equipment using the VAS/FACES scale to answer “How easy was it for you/your child/your patient to use the VR equipment?” A score of 0 represented “really easy to use,” and a score of 100 correlated with “really hard to use.” Lastly, participants were asked to use the VAS/FACES scale to assess the VR likeability by answering “How much would you/your child/your patient like to use VR for future IV procedures?” A score of 0 equated to “they would really like to use VR again” and 100 meant “they would really not like to use it again.”

### Statistical Analysis

This was a pilot feasibility study in a rare population. With insufficient background data on the overall feasibility of using VR technology in a clinical setting, a sample of 24 patients was selected to collect pilot data on the safety and usability, logistical issues of implementation, and to assess the durability and adaptability in the design of the equipment. The study was designed to be randomized and include a control SOC group to perform a preliminary test on the hypothesis that the length of time for IV procedures would be similar between the groups, but the sample size did not allow testing for equivalence. The justifications for this sample size were based on the rationale about feasibility, obtaining adequate precision on numerical estimates, and regulatory considerations [19]. This sample size allowed for evaluating the potential utility of the VR distraction technique on a wide range of ages and would result in parameter estimates that would aid in adequately powering future studies that would directly assess the benefits of VR in clinical settings.

Clinical data, including survey instrument data and adverse events, were entered into a hospital-compliant internet data entry system (REDcap) that included password protection and internal quality checks. Demographic data were captured from the patients’ electronic medical records. All demographic and safety data were described using summary statistics. To evaluate the hypothesis that the procedure time would be similar between the intervention and SOC groups, Mann-Whitney test was used. The length of IV procedure time was summarized by presenting mean and corresponding 95% CI as well as median and range. Kruskal-Wallis tests were applied to compare VAS/FACES scores given by the 3 rater groups as the secondary aim. Scores for the rater groups were summarized with medians and ranges. Statistical analyses were performed using the base R statistical package (R Foundation for Statistical Computing, Vienna, Austria).

## Results

### Demographics

**Figure 3:**
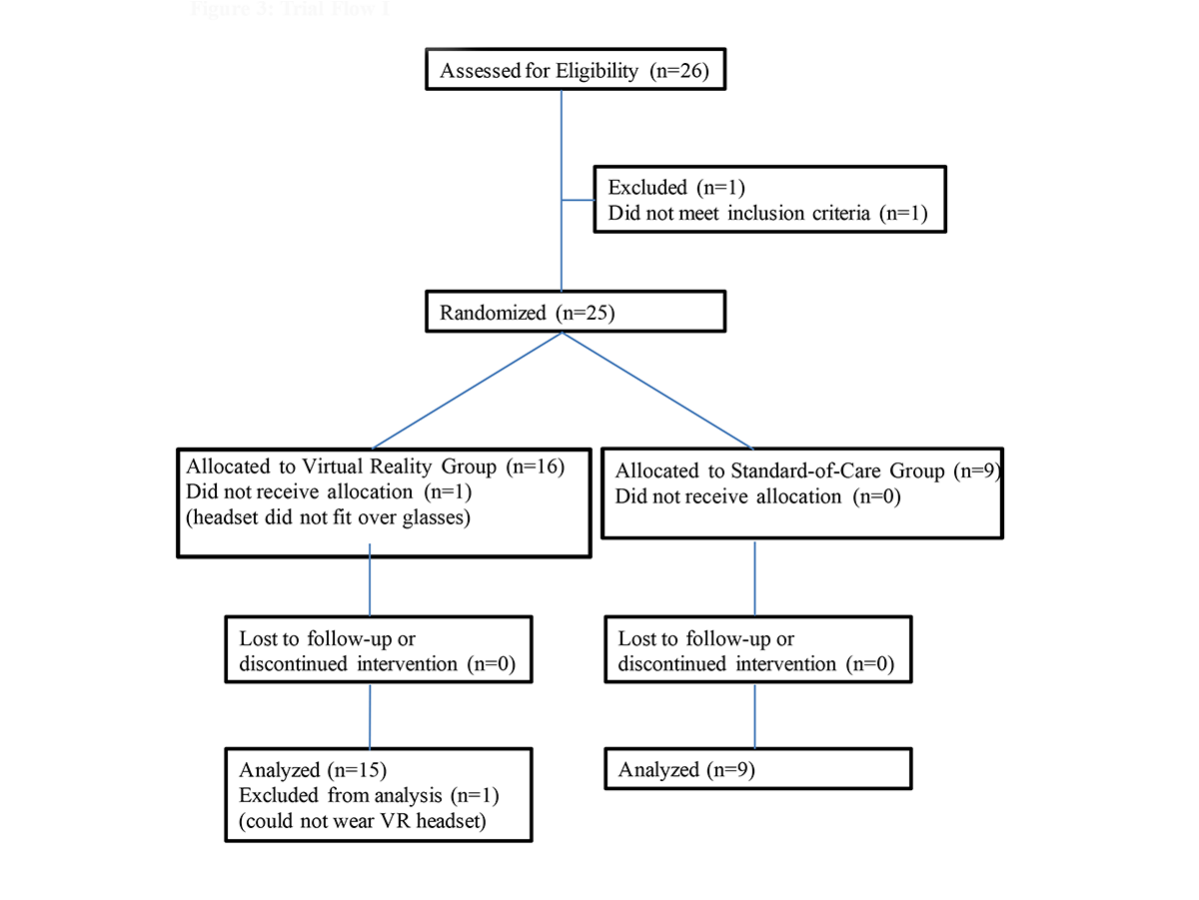
Trial flow diagram. VR: virtual reality.

In this study, 26 patients were screened for enrollment. There was 1 screen failure in a patient with moderately controlled seizures; therefore, 25 eligible patients aged 6-18 (median age, 13) years participated in study (Figure 3). In total, 16 patients were randomized to VR and 9 to SOC group; 1 patient in the VR arm was excluded from the analysis because of inability to wear the HMD over glasses. Of the remaining 24 patients, 83% (20/24) participants were males and 17% (4/24) were females; 54% (13/24) had HA and 46% (11/24) had HB, 42% (10/24) had severe hemophilia, 58% (14/24) had nonsevere hemophilia, and 54% (13/24) were on routine prophylaxis (Table 1). No patient harms or unintended effects were seen in either the SOC or VR group.

### Outcomes

#### Safety

No adverse events occurred during the trial. No patient experienced VR sickness, seizures, discomfort from the HMD, or infection-related events related to the VR experience.

#### Procedure Time

The median procedure time was 10 (range 1-31) minutes in the VR group, and was similar to 9 (range 3-20) minutes in the SOC group (*P*=.76; Table 1). The mean procedure time was 12.3 minutes (95% CI 7.2-17.4) in the VR group and 10.1 minutes (95% CI 5.7-14.6) in the SOC group.

#### Virtual Reality Equipment Usability

In this study, 60% (9/24) patients wore VR equipment during their entire procedure, 27% (4/24) utilized VR for part of their procedure, and 13% (2/24) only used VR prior to the procedure. Of the 4 participants who used VR for a part of their procedure, 1 removed the headset after the IV stick, while 3 used VR at the beginning of their procedures, removed the headset to watch the IV stick, and then resumed VR. The 2 participants, who only used VR prior to their procedures, chose to watch the entirety of the IV procedure. No technical issues were noted with the orchestration dashboard, headsets, or game hardware.

**Table 1:** Participants’ characteristics and median procedure times.

Variable		Virtual reality (n=15)	Control (n=9)	*P* value
Males, n (%)		12 (80)	8 (89)	.99
Median age, years		12.2	12.8	.56
**Hemophilia type, n (%)**				.99
	Hemophilia A	8 (53)	5 (56)	
	Hemophilia B	7 (47)	4 (44)	
**Hemophilia severity, n (%)**				.36
	Mild	3 (20)	3 (33)	
	Moderate	4 (27)	4 (44)	
	Severe	8 (53)	2 (22)	
Routine prophylaxis, n (%)		9 (60)	4 (44)	.68
Median procedure time, minutes		10	9	.76
Median orchestration events, n		17	N/A^a^	N/A

^a^N/A: not applicable.

**Table 2:** Median pre- and postprocedural visual analog/FACES scale score.

Question					Virtual reality	
**Nervousness prior to intravenous procedure (0=None, 100=Very)**						
				Nurse response		14
				Caregiver response		25
				Patient response		3
**Assessment of the distraction technique**						
	**Engaged or attentive (0=Yes it did, 100=Did not)**					
				Nurse response		13
				Caregiver response		13
				Patient response		18
	**Impact on anxiety (0=decreased, 100=increased)**					
			Nurse response			3
			Caregiver response			15
			Patient response			8
	**Impact on pain (0=lessened, 100=worsened)**					
			Nurse response			4
			Caregiver response			4
			Patient response			3
	**Likability (0=really like, 100=did not like)**					
			Nurse response			1
			Caregiver response			4
			Patient response			3
**Questions for the virtual reality group only**						
	**Ease of using the virtual reality equipment (0=Easy, 100=Hard)**					
			Nurse response			2
			Caregiver response			9
			Patient response			7
	**Virtual reality for future intravenous procedures (0=likely, 100=not likely)**					
			Nurse response			3
			Caregiver response			8
			Patient response			12

The VR equipment usability was favorably scored (0=easy to use, 100=hard to use) with median scores of 7, 9, and 2 by participants, guardians, and the nurse, respectively (Table 2). Additionally, patients, caregivers, and nurses positively rated the desire to use VR for future procedures on a scale of 0 (would really like to use VR again) to 100 (would really not like to use VR again); in majority (36/45, 80%) of evaluations, children, caregivers, and nurses reported that they would like to use VR for future procedures (score <50).

### Orchestration Events

The number of deliberate orchestration events per patient, including commands to enter or exit a mini-game, transport the player patient to a virtual location, connect to a paired iOS device, adjust the volume, open the video feed from the iOS device’s camera, or calibrate the headset units for fit and viewing preference, was available for 13 of the 15 patients. A server storage issue led to data loss, resulting in missing orchestration data for 2 patients. In the remaining 13 patients, there were a median of 17 orchestration events (range 7-28) per procedure.

### Nervousness or Worry and Pain

The groups did not differ statistically in preprocedural nervousness or worry, as rated by the participant (*P*=.67), caregiver (*P*=.37), or nurse (*P*=.27; Table 2). Median nervousness rating for the VR group was 3 (range 0-94) and 12 for the SOC group (range 0-100). Preprocedural patient nervousness did differ between age groups (*P*=.002) with those aged 6-12 years (median 50, range 0-100) being significantly more nervous compared with those aged 13-18 years (median 0, range 0-13). Preprocedural patient nervousness did not statistically differ between those on and not on prophylaxis (*P*=.64). Median nervousness rating was 6 (range 0-94) for those on prophylaxis and 27 for those not on prophylaxis (range 0-100). Both groups favorably viewed distraction techniques in terms of the effect on procedural anxiety, with median responses being 8 for the VR group and 10 for the SOC group (0=the distraction technique decreased anxiety and 100=the technique increased anxiety). Both VR and SOC distraction techniques had a positive influence on procedural pain (0=made pain better, 100=made pain worse); no statistically significant differences were observed between raters in the VR or SOC groups (Table 2).

### Level of Engagement

Scores of participants, nurses, and caregivers did not significantly differ between VR and SOC distraction techniques in terms of the ability to engage and hold attention (0=held attention and was engaging and 100=did not hold attention and was not engaging; Table 2).

## Discussion

### Principal Findings

This study represented the first known effort to translate VR from a research environment to a functioning pediatric clinic environment as a comprehensive platform including custom content (games), tools, and hardware (HMDs). The final platform included a high-tech, VR-based, child engaging distraction with child choice incorporated into a platform that allowed for need-based adult involvement. The final HMDs were multisized, lightweight, and disposable and had an estimated unit cost of less than US $55. If manufactured at scale, the estimated cost of the headphones, headsets, lenses, cardboard, elastic, and hooks and loops is less than US $20. HMDs accommodated 2 distinct size faces—one for average face size of patients over 15 years of age and another for that of patients under 15 years of age. The game navigation was hands-free and wireless. Games produced were designed to appeal to a wide age range of male and female patients. Games were also engineered to achieve a consistent 30 frames per second playback rate on limited resource devices. Additionally, games afforded the player control over a child engaging gameplay environment without requiring the use of the patients’ hands—a unique affordance ingrained in this platform. The system achieved hands-free gameplay via the introduction of proprietary hardware in the custom HMD, which is capable of monitoring the breathing of the play and providing this feedback to the games. The system did not require a high-end computer or technical expertise for installation, and the orchestration features allowed for customization to patient needs and family engagement. Successful incorporation into the clinic was demonstrated by the lack of safety and technical issues. Although our sample size did not allow equivalence comparison, we had similar lengths of procedure times in both groups.

The games themselves mitigated the risk of the player developing simulator sickness with the standard technique of introducing a static frame of reference during periods of motion. While static frames of reference are a conventional technique used to prevent VR sickness, the technique’s manifestation in our system was novel in approach in that the technique utilized world elements as the static frame of reference. This execution of the technique, therefore, did not sacrifice the immersive quality of the environment. In addition, the static frames introduced a form of kinesthetic reinforcement for the players’ physical sensation to the virtual world, thus lending to their immersive quality. For example, in the snorkeling game in the game world, players observed a projection of a virtual diving mask in their game view. The projection not only acted as a static frame of reference that thematically fits within the game environment but also created a relationship between the physical sensation of the physical HMD and the virtual mask as seen in the game world.

Our environments incorporated a core component of distraction theory most significantly, with affordance of control and choice as a means to distract [28]. This method is inherent in child engaging games, with our platform bringing this method to the patient population through hands-free game controls and thus representing the first set of pediatric-focused games, which utilize this technique while not requiring hand movement to interact and thus bringing this technique via games to patients receiving IV procedures. In total, the techniques employed throughout our ecosystem resulted in games that apply distraction theory through child engaging VR environments while not triggering simulator sickness in participants, as demonstrated in the collected data.

Pain and anxiety related to procedures is a concern for patients, families, and providers, particularly in pediatric settings. Because children with hemophilia have frequent needle procedures, they represent a population that could really benefit from VR-based distraction. While there is strong evidence for the success of distraction during pediatric procedures, it remains unclear which of the 4 main elements of effectiveness (high vs no or low tech, child engaging vs passive, degree of adult involvement, and availability of child choice) contribute to the success of the technique [9,10]. Encouragingly, 87% of participants wore the VR equipment during some or all of their procedure, and overall scores regarding the impact of VR on pain, anxiety, usability, likeability, and level of engagement were favorable.

### Limitations

The trial was limited by small sample size and single-institution design. Additionally, we studied only a single intervention, so we were unable to test if the VR platform would continue to perform well with future use in the same patient. The study was underpowered to evaluate the equivalence of procedure time. The study was also underpowered to compare VR versus SOC attributes, but the majority of caregivers and providers had favorable ratings of VR, and 80% of participants, all of whom had previous experience with SOC distraction in our clinic, reported a desire to use VR for future procedures. The outcomes related to pain and anxiety were self-reported. Addition of objectives measures of distress, such as blood pressure and heart rate, would strengthen future studies.

### Conclusions

We demonstrated that a custom-designed VR platform could be used safely during IV procedures in a pediatric hemophilia population with specific design needs. These results warrant future exploration to assess the impact of our platform on its ability to reduce the burden of IV procedures on patients, parents, and clinical care providers. Future studies will be needed to validate our findings in other disease populations, clinical settings, and institutions with larger participant numbers. While patients with hemophilia may have more IV experiences than most children, it is likely that the positive effects of high-quality distraction would be generalizable to IV procedures in any pediatric population. We plan larger studies comparing (1) our child engaging platform versus a passive distracter; (2) the degree to which adult involvement impacts successfulness; (3) the importance of the availability of child choice in distracters; (4) ability to decrease procedural chemical sedation; and (5) cost. Additionally, nonrandomized trials allowing children to choose their distraction technique of choice during procedures would generate useful information. The inclusion of objective measures of pain and anxiety would strengthen future studies. This study suggests that a custom VR ecosystem with clinician orchestration is a promising modality to provide distraction during IV procedures in pediatrics with the potential to mitigate the perception of procedural pain and anxiety.

## Appendix

Multimedia Appendix 1 Voxel Bay video experience.

Multimedia Appendix 2 CONSORT-EHEALTH checklist (V 1.6.1).

## Abbreviations

HA: hemophilia A
HB: hemophilia B
HMD: head-mounted display
HTC: Hemophilia Treatment Center
IV: intravenous
SOC: standard of care
VAS: visual analog scale
VR: virtual reality
